# Analysis of simultaneous space-time clusters of *Campylobacter *spp. in humans and in broiler flocks using a multiple dataset approach

**DOI:** 10.1186/1476-072X-9-48

**Published:** 2010-09-22

**Authors:** Malin E Jonsson, Berit Tafjord Heier, Madelaine Norström, Merete Hofshagen

**Affiliations:** 1 National Veterinary Institute, Department for Health Surveillance, POB 750 Sentrum, 0106 Oslo, Norway; 2 Norwegian Institute for Public Health, POB 4404 Nydalen, 0403 Oslo, Norway

## Abstract

**Background:**

Campylobacteriosis is the most frequently reported zoonosis in the EU and the epidemiology of sporadic campylobacteriosis, especially the routes of transmission, is to a great extent unclear. Poultry easily become colonised with *Campylobacter *spp., being symptom-less intestinal carriers. Earlier it was estimated that internationally between 50% and 80% of the cases could be attributed to chicken as a reservoir. In a Norwegian surveillance programme all broiler flocks under 50 days of age were tested for *Campylobacter *spp. The aim of the current study was to identify simultaneous local space-time clusters each year from 2002 to 2007 for human cases of campylobacteriosis and for broiler flocks testing positive for *Campylobacter *spp. using a multivariate spatial scan statistic method. A cluster occurring simultaneously in humans and broilers could indicate the presence of common factors associated with the dissemination of *Campylobacter *spp. for both humans and broilers.

**Results:**

Local space-time clusters of humans and broilers positive for *Campylobacter *spp. occurring simultaneously were identified in all investigated years. All clusters but one were identified from May to August. Some municipalities were included in clusters all years.

**Conclusions:**

The simultaneous occurrence of clusters of humans and broilers positive for *Campylobacter *spp. combined with the knowledge that poultry meat has a nation-wide distribution indicates that campylobacteriosis cases might also be caused by other risk factors than consumption and handling of poultry meat.

Broiler farms that are positive could contaminate the environment with further spread to new broiler farms or to humans living in the area and local environmental factors, such as climate, might influence the spread of *Campylobacter *spp. in an area. Further studies to clarify the role of such factors are needed.

## Background

Campylobacteriosis is the most frequently reported zoonosis in the EU [1]. Between 2002 and 2008 the incidence for domestically acquired campylobacteriosis in Norway varied between 19.1 (2002) and 26.2 (2005) reported cases per 100 000 inhabitants [2]. Outbreaks of campylobacteriosis are relatively rare; most infections seem to be sporadic [3]. However, the epidemiology of sporadic campylobacteriosis, especially the routes of transmission, is to a great extent unclear [4,5]. Internationally it is estimated that handling, preparation and consumption of broiler meat may account for 20% to 30% of the human cases [6]. However, it has been suggested that chicken as a reservoir might account for between 50% and 80% of the cases [6]. The latter estimates are based on studies of sequence typing of *Campylobacter *and the assumption that strains from chicken may reach humans by other pathways than food, e.g. through the environment or by direct contact. Poultry easily become colonised with *Campylobacter *spp. being symptomless intestinal carriers of the organism. Once some broilers in a flock become colonised, the bacteria spread rapidly to most of the birds in the flock [7,8]. Several epidemiological studies have identified management and environmental risk factors for the occurrence of *Campylobacter *spp. in broiler flocks, e.g. low level of bio-security, number of broiler houses on a farm, drinking water quality, season and the presence of other farm animals in the vicinity [9-16]. Norway implemented an Action Plan against *Campylobacter *spp. in broilers in 2001, hereafter called the Action Plan, consisting of three parts: a surveillance programme including all commercial Norwegian broiler flocks slaughtered before 50 days of age (virtually all broiler flocks), a follow-up advisory service for farms delivering positive flocks and surveys of broiler meat products at the retail level [17]. In the surveillance programme during the period 2001-2007, each flock was tested for *Campylobacter *spp. in caecal droppings 4-8 days before slaughter and again at slaughter at the abattoir as described elsewhere [15,18]. In this period the annual incidence of Norwegian broiler flocks that tested positive for *Campylobacter *spp. varied between 3.3% (2004) and 6.3% (2002) with considerable differences between seasons and regions.

The aims of the current study was to identify simultaneous local space-time clusters each year from 2002 to 2007 for human cases of campylobacteriosis and for broiler flocks testing positive for *Campylobacter *spp. using a multivariate spatial scan statistic method and to obtain the relative risk of being a case within a such cluster. Identification of clusters could indicate a possible presence of common factors associated with the dissemination of *Campylobacter *spp. for both humans and broilers.

## Materials and methods

### Study period and study area

The study period was between 1 January 2002 and 31 December 2007. The study area included all municipalities in Norway with a broiler population using the centroid for each municipality as the geographical location. The number of municipalities in the study area varied between 110 and 119 per year (Table 1).

**Table 1:** Multivariate SaTScan™ analyses of *Campylobacter *spp. in humans and broilers, 2002-2007 in Norway

Year	**No. muni.**^**#**^	No. cases		Proportion cases in cluster (%)		Cluster	Observed cases (expected cases)		**No. muni.**^**# **^**in cluster**	RR^*^		*p*-value	Time span
		***Hu***^***#***^	***Br***^***#***^	***Hu***^*#*^	***Br***^*#*^		***Hu***^**#**^	***Br***^**#**^		***Hu***^**#**^	***Br***^**#**^		
2002	119	343	174	9.0	19.0								
						1	4 (0.5)	15 (0.4)	3	7.6	46.1	<0.001	3 July - 30 July
						2	13 (0.9)	4 (0.5)	5	14.3	7.6	<0.001	12 June - 9 July
						3	1 (0.1)	5 (0.1)	1	8.1	63.7	<0.001	10 July - 30 July
						4	11 (1.3)	2 (0.6)	6	8.9	3.2	<0.001	19 June - 16 July
						5	2 (0.7)	7 (0.4)	4	3.0	17.1	0.006	10 July - 6 Aug.
2003	117	378	145	7.1	15.9								
						1	18 (1.4)	22 (0.6)	7	13.5	39.4	<0.001	3 July - 30 July
						2	9 (0.7)	1 (0.4)	4	13.0	2.5	0.004	26 June - 16 July
2004	117	396	99	2.5	5.0								
						1	10 (1.5)	5 (0.3)	6	6.9	17.0	<0.001	24 July - 20 Aug.
2005	114	418	114	10.5	13.2								
						1	17 (1.6)	8 (0.4)	6	10.9	20.1	<0.001	26 June - 23 July
						2	18 (1.8)	2 (0.8)	7	10.1	2.5	<0.001	17 July - 13 Aug.
						3	9 (1.1)	5 (0.4)	5	8.3	13.5	<0.001	31 July - 27 Aug.
2006	112	421	157	2.6	21.7								
						1	2 (0.9)	17 (0.4)	4	2.1	42.9	<0.001	17 July - 13 Aug.
						2	5 (0.2)	5 (0.1)	1	22.4	54.6	<0.001	17 July - 13 Aug.
						3	1 (0.2)	6 (0.1)	1	4.5	64.2	<0.001	17 July - 13 Aug.
						4	3 (0.6)	6 (0.3)	3	5.4	19.8	0.005	21 Aug. - 10 Sep.
2007	110	392	197	19.6	21.8								
						1	3 (0.6)	20 (0.4)	3	4.8	51.3	<0.001	26 June - 23 July
						2	26 (1.8)	5 (1.5)	8	15.3	3.3	<0.001	29 May - 25 June
						3	24 (1.7)	5 (1.0)	7	14.6	5.0	<0.001	19 June - 16 July
						4	11 (0.9)	4 (0.5)	6	12.7	7.7	<0.001	17 July - 6 Aug.
						5	1 (0.2)	5 (0.1)	1	5.0	41.1	0.003	19 June - 16 July
						6	2 (0.1)	3 (0.1)	1	19.7	46.0	0.030	24 July - 6 Aug.
						7	10 (1.2)	1 (0.6)	4	8.2	1.8	0.038	10 July - 6 Aug.

^* ^RR = The relative risk of being a human case or a broiler case within a space-time cluster compared to outside.

^#^muni. = municipalities, Hu = human, Br = broiler

Characteristics of simultaneous space-time clusters of human campylobacteriosis and broiler flocks positive for *Campylobacter *spp. identified by the multivariate SaTScan™ Poisson model. The clusters are ranked according to their likelihood ratio test statistic.

### Broiler population

The target population was commercial broiler flocks in Norway. Broiler flock was the study unit and data on broiler flocks with corresponding *Campylobacter *spp. results was generated from the Action Plan. The geographical location for each farm defined on the municipality level was obtained from the Agricultural Property Registry (the official census database for information on agricultural properties in Norway). In the Action Plan there were 709 farms that delivered 22 281 broiler flocks during the study period. Farms which had delivered only one or two flocks for slaughter in only one of the years (n = 63) and farms where geographical coordinates were missing (n = 23) were excluded. In addition, to avoid inclusion of a flock more than once, only the first flock result per farm in a seven-day period was included (2559 flocks excluded). This resulted in a study population of 19 484 broiler flocks originating from 623 different farms.

**Figure 1:**
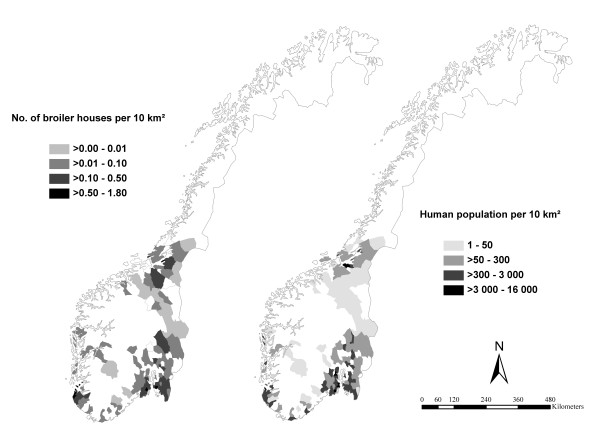
**Human population density and broiler house density per municipality in Norway in 2004**.

Broiler house density for each municipality and year was calculated using the number of broiler houses as the numerator and area data for each municipality, which was obtained from the Norwegian Mapping Authority, as the denominator (Figure 1).

### Human population

There were between 1.57 (2007) and 1.63 (2002) million inhabitants in the study area each year during the study period. The human population figures were obtained from Statistics Norway. The human population density per municipality each year was calculated using the human population figures as the numerator and area data of the municipalities as the denominator (Figure 1).

### Case definitions

A broiler case was defined as a broiler flock where at least one of the test results was positive for *Campylobacter *spp. The date of slaughter of a positive broiler flock was defined as the time of the occurrence of a case. In total, 886 broiler cases were included. The number of broiler cases each year is shown in Table 1. Data on confirmed domestic cases of human campylobacteriosis (n = 2348) within the study area during the study period was obtained from the Norwegian Surveillance System for Communicable Diseases (MSIS, Norwegian Institute for Public Health) (Table 1). A case was defined as a person with laboratory-confirmed *Campylobacter *spp. in faeces. The reported day of sampling was used as the time of occurrence of a case. The municipality in which the infection was supposed to have occurred was used as the geographical location of a case. The information of the geographical location was not available for 27% of the cases, and for those the municipality of residence was used as the geographical location.

### Statistical and space-time analyses

Statistical data handling and analyses were performed using SAS Enterprise Guide version 4 for Windows (SAS Institute Inc., Cary, NC, USA) and STATA version 10 (Stata Corp., College Station, TX, USA).

Space-time cluster analysis was performed each given year for both human and broiler cases in the study area using the space-time scan statistic SaTScan™ v.8.2.1 software (Kulldorff M. and Information Management Services, Inc. SaTScan™ v8.2.1 http://www.satscan.org/) [19]. The method is based on cylinders, or scanning windows, centred on each geographical location, with the base representing space and the height representing time. The alternative hypothesis assumes that there is an elevated risk within the window compared to outside. The window with the maximum likelihood constitutes the most likely cluster and this is the cluster least likely to have occurred by chance. In addition, secondary clusters are identified, that are ordered according to their likelihood ratio test statistic. *P*-values are obtained through Monte Carlo hypothesis testing. Significant clusters (*p *< 0.05) and relative risks, comparing the risk of being a case inside the window with the risk of being a case outside, are calculated.

The analysis was performed using the Poisson model, which requires case and population counts for each geographical unit as well as the geographical coordinates. To identify clusters with high power occurring simultaneously in humans and broilers, the multivariate scan statistic analysis approach was used where adjustment for the different informational content when combining different datasets is automatically performed as described by Kulldorff et al. [20]. In this method the log likelihood ratios are calculated for each of the included datasets and are then summed up. The sum constitutes the likelihood for a particular window. The window with the maximum sum of likelihood ratio is the most likely cluster. It was assumed under the null-hypothesis that the number of human cases and broiler cases are proportional to the population of humans and broilers respectively in each municipality. Each year of the study period was analysed separately. The size of the scanning window was allowed to increase to a maximum radius of 50 km and maximum time of 30 days and cases were aggregated into intervals of seven days. The maximum time was based on the length of the rearing period for broilers that was approximately 30 days.

To identify clusters not caused by high population density, a Poisson regression analysis was performed on each of the datasets to obtain the expected number of cases. For human cases, human population density was included in the model and for broiler cases broiler house density was included. The expected numbers of cases were used as the population counts in the SaTScan™ models.

To test for statistical significance, 999 replications of Monte Carlo hypothesis testing were conducted. The level of significance was set to *p *≥ 0.05. The analysis was adjusted to only include clusters with cases present simultaneously in both datasets. To identify areas where clusters were present recurrently during the years, the municipalities included in clusters were aggregated by year. In addition, the proportion of human cases located inside a cluster, as well as the proportion of broiler cases located within a cluster each year, was calculated. The locations of the clusters was visualised with ArcGIS 9.2 (ESRI, Redlands, CA, USA).

## Results

### Descriptive analyses

**Figure 2:**
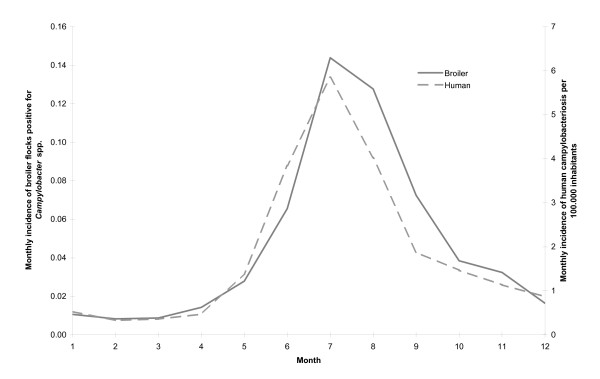
**Mean monthly incidence of *Campylobacter *spp. in humans and broilers**. Mean monthly incidence of human campylobacteriosis and of broiler flocks positive for *Campylobacter *spp. 2002-2007 in Norway.

Each of the 623 broiler farms had between one and six broiler houses. The majority of farms had only one broiler house, varying between 87.5% and 89.9% for single years. In Norway, the broiler production is concentrated in five distinct areas where the density of broiler houses is rather high. On the municipal level, the 5-percentile and the 95-percentile of the number of broiler houses per municipality were 1 and 14, respectively, for all years. Figure 1 shows broiler house density (number of houses per 10 km^2^) per municipality in 2004. The densities showed few changes from year to year, thus the maps for each year are not shown. The 5-percentile and the 95-percentile of human population density per municipality in the study area in 2004 were 16 and 3421 inhabitants per 10 km^2^, respectively (Figure 1). The mean monthly human incidence of campylobacteriosis and the mean monthly incidence of broiler flocks positive for *Campylobacter *spp. 2002-2007 are shown in Figure 2. Both peak during the summer months.

### Statistical and space-time analyses

**Figure 3:**
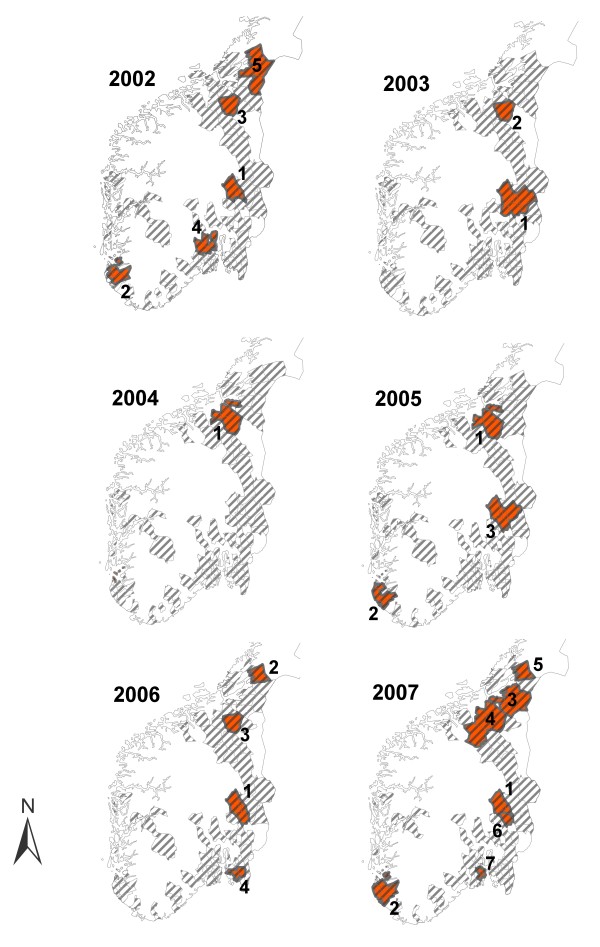
**Simultaneous space-time clusters of *Campylobacter *spp. in humans and broilers**. Geographical location of simultaneous space-time clusters of human campylobacteriosis cases and broiler flocks positive for *Campylobacter *spp. each year 2002-2007 in Norway. Clusters were identified by the multivariate SaTScan™ Poisson model. The shaded areas indicate the study area during different years (municipalities with broiler farms). The clusters are ranked according to their likelihood ratio test statistic.

**Figure 4:**
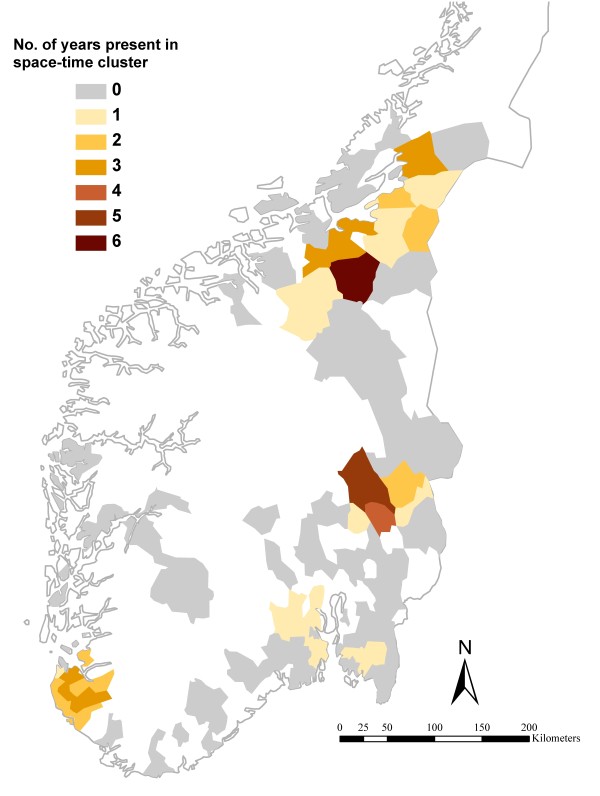
**Frequency of inclusion in space-time clusters of *Campylobacter *spp. in humans and broilers**. The numbers of years the municipalities were included in a simultaneous space-time cluster of human campylobacteriosis cases and broiler flocks positive for *Campylobacter *spp. each year 2002-2007 in Norway. Clusters were identified by the multivariate SaTScan™ Poisson model.

Between one (in 2004) and seven (in 2007) statistically significant space-time clusters were identified each year (Table 1). The relative risk of being a human case or a broiler case within a space-time cluster compared to outside varied from 2.1 to 22.4 and from 1.8 to 64.2, respectively as shown in Table 1. All clusters except one occurred during the summer months (from 29 May until 27 August) and most clusters had a time span of 27 days. The locations of the clusters are shown in Figure 3. A total of 44 (34.6%) different municipalities with broiler populations were included in a space-time cluster from one to six times during the study period (Figure 4). Between 2.5% and 19.6% of the human cases located in the study area were included in a space-time cluster during different years.

## Discussion

The current results suggest that space-time clusters of *Campylobacter *spp. in man and in broiler simultaneously occur in all of the investigated years. They occur almost only in the summer months and in certain parts of Norway more frequently than in others. In Norway, poultry slaughterhouses are few and large scale. Poultry meat originating from one farm is widely distributed throughout the country. Occurrence of local production of poultry meat, where the poultry are raised, slaughtered, retailed and consumed in the same area, is negligible and thereby the vast majority of human cases of campylobacteriosis will certainly not be caused by consumption of broiler meat from neighbouring farms. Thus, the results in the present study indicate that there could be local factors, some possibly common for man and broilers, which prompt the occurrence of human campylobacteriosis and of *Campylobacter *spp. colonisation in broilers in specific areas. In other countries it has been demonstrated that the same sequence types (ST's) to a large extent are found in both man and in broiler [5,21]. On the other hand, Levesque et al. found, when investigating clonal complexes of *Campylobacter jejuni *from different sources, that sporadic *Campylobacter jejuni *infections in man may frequently arise from sources other than broilers [22]. Sequence typing has not been performed on Norwegian strains. It would be interesting to investigate the geographical dissemination of different ST's combined with information on sources. However, some research has been done where geographical differences of ST's from sheep and cattle and from a rural environment have been investigated [23,24]. A possible identification of the same ST's in man and in broiler in the same geographical area could be related to external factors acting as reservoirs or vectors/vehicles for dissemination. Broiler flocks that are positive might contaminate the environment, particularly if untreated litter is deposited outside. In a Norwegian study, it was shown that a *Campylobacter *spp. subtype found in one broiler flock was also found in a neighbouring flock some weeks later [25]. In Denmark it was shown that flies adjacent to the broiler house could be contaminated with *Campylobacter *spp. and that large number of flies had access to the broiler house via the ventilation system, suggesting that flies could be a vector/vehicle for transmission between farms [26]. It has also been suggested that the incidence of human campylobacteriosis was higher in populations living in rural and agricultural areas, with the highest rates occurring in populations living in proximity to high densities of farm animals [27,28] and in a recent EFSA opinion on the quantification of the risk posed by broiler meat to human campylobacteriosis, it was stated that broiler meat might account for 20% to 30% of human campylobacteriosis, while chicken as a reservoir might account for 50% to 80%, for instance by strains from chicken contaminating drinking water, crop land or lakes [6]. In Norway, up to 19.6% of the human cases in broiler areas were located within a space-time cluster yearly, indicating there are factors other than broilers that are important in contributing to sporadic campylobacteriosis in these areas. In the current study, the observed patterns may be influenced by factors acting on both global and local scales. In some areas clusters occurred every year, suggesting that factors acting on a local scale were involved. Such factors could be climate, landscape or geography. Human campylobacteriosis and colonisation of broilers in Norway, as in other European countries, follow a seasonal pattern with a peak in the summer [17,29] strongly suggesting that climate plays a role. Variation in climate was previously described as having an effect on the occurrence in both broilers and man [29,30]. However, more knowledge about the impact of climate is needed.

The sensitivity of the sampling of broiler flocks in the Action Plan was not estimated, but with the practice of two samples per flock at different time points, high within-flock prevalence and a sampling regime in accordance with the EU Baseline survey on *Campylobacter *spp. in broiler flocks, the flock-level sensitivity of the sampling is thought to be high. The aim of the Action Plan was to identify most possible flocks colonised with *Campylobacter *spp., to be able to effectuate interventions such as deep freezing or heat treatment of the carcasses when a positive flock was identified.

When studying phenomena that occur in different populations where there might be common environmental or other sources, analysing clusters that occur simultaneously in all populations could assist in generating a hypothesis of such sources. In the current study, an approach in SaTScan™, where it was possible to analyse multiple datasets simultaneously without combining the datasets beforehand was used [20]. As the analyses were performed for both space and time, it would probably be very few clusters that overlapped exactly in space and time if the analyses were performed univariately. In addition, Kulldorff et al [20] claim that there could be loss of power if the datasets are analysed univariately.

Though most cases of campylobacteriosis are sporadic, some local outbreaks are identified in Norway every year, probably most often with a point source of infection, e.g. drinking water. The epidemiology of outbreaks might be different than that of sporadic campylobacteriosis. In the current study some significant clusters were identified where only human cases and not broiler cases were present. It was assumed that local outbreaks of human campylobacteriosis were causing high log likelihoods without contribution of the log likelihoods from the broiler cases. These clusters were not included in the results because the objective was to investigate simultaneous clusters of *Campylobacter *spp. in both humans and in broilers. Because only one third of the human population was included in the analyses, some clusters due to outbreaks were not detected. One example is the large Røros outbreak of campylobacteriosis in May 2007 [31], where Røros municipality did not have a broiler population and therefore was not included in the study. In addition, campylobacteriosis is probably, as is the case with most acute infectious enteric diseases, highly underreported. If there was a geographically skewed distribution of cases, for instance if there was geographical variation in how prone people were to visit health care providers during an incidence of a diarrhoeal disorder or how thorough health care personnel were in collecting samples from patients with such symptoms, this could have led to biased results by over- or underestimating the number of cases in different geographical areas.

## Conclusions

In areas with a broiler population, simultaneous local space-time clusters of *Campylobacter *spp. cases in humans and in broilers were identified in all investigated years. The occurrence of such clusters combined with the knowledge that poultry meat has a nation-wide distribution indicates that campylobacteriosis cases might also be caused by other risk factors than consumption and handling of poultry meat.

Broiler farms with *Campylobacter *spp. positive flocks could contaminate the environment with further spread to new broiler farms or to humans living in the area and local environmental factors, such as climate, might influence the spread of *Campylobacter *spp. in an area. Further studies to clarify the role of such factors are needed.

## Competing interests

The authors declare that they have no competing interests.

## Authors' contributions

The study was designed by all authors. MEJ and MN performed the statistical analyses and the analyses of the results was conducted by all authors. MEJ drafted the manuscript and all authors revised, read and approved the final manuscript.
